# Patients' Views and Expectations of Consultations in Primary Care in Malaysia: A Qualitative Study

**DOI:** 10.7759/cureus.86553

**Published:** 2025-06-22

**Authors:** Muhammad Hanif Bin Omar, Nurul'Ain Binti Hamdan, Afif Bin Mohd Amirruddin, Nor Nabilah Binti Azmi

**Affiliations:** 1 Family Medicine, Klinik Kesihatan Karak, Karak, MYS; 2 Family Medicine, Klinik Kesihatan Dato' Keramat, Kuala Lumpur, MYS; 3 Family Medicine, Klinik Kesihatan Dengkil, Dengkil, MYS; 4 Family Medicine, Klinik Kesihatan Beranang, Beranang, MYS

**Keywords:** general practice, global healthcare systems, non-communicable disease, patient's expectations, patients’ perceptions, physical consultation

## Abstract

Introduction: The increased prevalence of noncommunicable diseases (NCDs) in Malaysia has strained its public health services. Lifestyle changes remain a critical part of the management of NCDs. Primary care doctors are expected to provide lifestyle advice during consultations. Perception and expectations of these consultations directly affect the health-modifying behaviours of patients and the outcome. Understanding patients’ expectations in various contexts helps in tailoring good and effective consultations. The Health Belief Model (HBM) is a theoretical framework widely used to predict health behaviours. The authors hypothesise that a good doctor’s consultation should cover all six domains of HBM to trigger patients to change their health behaviour.

Methods: This was an exploratory qualitative study using semi-structured interviews with patients recruited using purposive sampling, at three different government health clinics in the Hulu Langat district of Selangor, Malaysia. Data were analysed deductively utilising HBM as the theme for patients’ views, and an inductive approach for patients’ expectations.

Results: Consultations positively influenced participants’ health behaviour. However, the perception of their NCD severity was found to be superficial. Self-efficacy in sustaining changes was a notable barrier. Expectations included comprehensive health advice, empathy, and professionalism. There is a subset of patients who had no expectations coming into these consultations.

Conclusion: Consultations were perceived to improve health behaviour but lack depth in understanding severity and self-efficacy. Patients expect comprehensive, empathetic, and professional consultations. Some had no expectations, which may reflect passivity or trust.

## Introduction

Noncommunicable diseases (NCDs) are prolonged conditions that manifest as a result of a complex interplay of genetic, physiological, environmental, and behavioural determinants. Vulnerabilities to NCDs are multifaceted. Unhealthy dietary patterns, sedentary lifestyle, excessive alcohol consumption, and tobacco use can lead to metabolic changes that could significantly increase a person's risk of developing NCDs and their complications over their lifetime [1].

According to the National Health and Morbidity Survey 2023 by the Ministry of Health, Malaysia, NCDs were found to be the main cause of death and disability among Malaysians. Over half a million adults in Malaysia live with at least four NCDs, while another 2.3 million adults were found to be living with three [2]. The growing prevalence of NCDs in Malaysia has placed an increased strain on its public health system through increased demand for healthcare services. This presents an urgent need for effective preventive strategies and interventions to mitigate the impact of NCDs on the Malaysian population's health. In most instances, primary care doctors in government health clinics are often the first point of contact for NCD patients in Malaysia. They are the patient’s primary source of information about their diseases, as well as interventions, either pharmacological or lifestyle modifications. Hence, the doctor-patient consultation is a fundamental aspect of primary healthcare, serving as a critical point of interaction between healthcare providers and patients.

Moreover, evidence suggests the doctor-patient relationship is key in facilitating behaviour change conversations. Consequently, effective communication, tailored information, and rapport building are essential for successful behaviour change interventions in primary care, leading to better health outcomes [3]. Studies have found that a mismatch in patient expectations and actual experience during a doctor’s consultation may lead to patients’ dissatisfaction, potentially leading to worse health outcomes [4]. Therefore, there is a need to explore these factors in the local context. Research has highlighted that good communication between the doctor and the patient increases trust, which may positively impact compliance with treatment plans and health outcomes [5]. Mismatches in vital components of communication between a doctor and patient can lead to loss of trust and satisfaction [6].

While a good doctor-patient relationship remains the most important expectation of patients from their doctors, there could be other nuances found in different geographical and demographical contexts. Comprehending these perspectives could play a pivotal role in constructively changing patients’ healthcare-seeking behaviour, adherence to medical advice, and ultimately improving overall health outcomes.

In the 1950s, Hochbaum et. al. developed the Health Belief Model (HBM) to understand the social dynamics of communities failing to participate in disease prevention programs [7]. This framework posits the decision-making process individuals undergo regarding their health. According to this model, there are six key factors that can influence an individual's actions towards their health: perceived susceptibility to illness, perceived severity of the illness, perceived benefits of taking preventive measures, perceived barriers to taking action, self-efficacy in executing the recommended health behaviour, and cues to action [8]. From predicting preventive health behaviours, HBM has since evolved into various applications in research, ranging from comprehending health promotion behaviours to compliance behaviours. Because of this, HBM was chosen as the theoretical model for this study as it has repeatedly demonstrated its relevance, applicability, adaptability, and effectiveness across various cultural contexts and health domains worldwide, and remains one of the most widely used applied theories of health behaviour. 

A meta-analysis on the effectiveness of lifestyle intervention in the Southeast Asia region has proven that lifestyle modifications are effective in dampening the effects of NCDs [9]. The authors of the current study, therefore, hypothesise that a good doctor’s consultation should cover all six domains of HBM to trigger patients to change their lifestyle based on the doctor’s medical advice. This qualitative research objective is to explore and understand patient perspectives and expectations regarding the effectiveness of their doctor's consultations in primary care, and how the consultations have changed their health behaviour, using HBM as the theoretical framework.

## Materials and methods

This was a qualitative study conducted in the Hulu Langat district in Selangor, Malaysia. It is the fourth largest and seventh most dense district in Malaysia [10]. It consists of a mix of urban and rural settlements with a demographics similar to the demographics of all of Malaysia. Three out of 14 government health clinics in the district were chosen: Klinik Kesihatan Kajang, Klinik Kesihatan Bandar Tun Hussein Onn, and Klinik Kesihatan Bangi, which share similar patient demographics and socioeconomics, capacity, size, attendance, and are located at suburban locations to ensure homogeneity of data collected. In addition to that, they have their own NCD units where the doctors only see NCD cases. The study was approved by the Medical Research & Ethics Committee, Ministry of Health, Malaysia (approval number: NMRR ID-23-02158-B56 (IIR) dated August 30, 2023).

Conceptual frameworks and study design

An exploratory qualitative approach was utilised to emphasise conceptualising and interpreting patients’ views and expectations of doctors’ consultations with regard to their health behaviour changes [11]. This was guided by the HBM as the theoretical framework, which was also used as the basis of the interview guide (see Appendices). This provided a structured approach to understand the various experiences of the participants, while retaining the flexibility of identifying new themes during the analysis of the data gathered.

An exploratory qualitative approach was chosen as researching views and expectations regarding doctors’ consultations was found to be difficult to objectify, as the concepts were broad and multidimensional. Quantitative studies do not translate the emotional components of what a patient goes through during a consultation. There is a wide array of processes that affect these moments, leading to variabilities in content and, to an extent, response towards these consultations. A qualitative study helps by producing different perspectives that are further exceeds what quantitative research can bring.

The authors adopted criteria described by Lincoln and Guba [12] to maintain the credibility, transferability, dependability, and conformability of qualitative work. Several strategies ensured study rigor: (i) meticulous documentation covering all visits, interviews, and participant interactions, (ii) use of an updated electronic codebook, and (iii) collaborative analysis of interviews by the research team. Additionally, interviewers clarified any discrepancies uncovered during analysis.

Sampling

Purposive sampling was used, aiming for maximum variation across age, sex, race, and medical conditions of the participants, and participants were recruited until data saturation was achieved, when no additional data were found and similar instances and findings were found repeatedly. This was to focus on transferability rather than generalisability, with an aim of theoretical completeness.

Eligibility criteria

Malaysian patients proficient in Bahasa Melayu or English, aged above 18 years, who visited the NCD unit for follow-up were included. Patients visiting the NCD unit* *for the first time and those with disability or who needed an interpreter when communicating were excluded.

Data collection

One-to-one in-depth interview (IDI) using a semi-structured interview guide was chosen as the data collection tool rather than focus group discussions (FGD), as the objective was to explore personal experiences, rather than a community experience of doctors’ consultations. IDIs also ensure the confidentiality of participants' medical conditions. Written consent was obtained prior to the interviews. An interview guide (see Appendices) was created based on the HBM, with specific prompting questions to elicit participants' recent experiences with their doctor’s consultation. 

Pilot interviews were done with three different participants during the development of the guide in December 2023. The pilot validated the relevance and clarity of the questions posed, and it provided essential training in the interview process, including the formulation of probing questions as well as improving the flow of the interview. This ensured that the subsequent interviews conducted were effective, yielding rich, qualitative data that has significantly contributed to the robustness and reliability of the research findings.

Interview sessions were conducted by the research team members, who had never worked at the clinics before and did not know any of the participants, to avoid potential response bias. Each interview was conducted between December 2023 and February 2024 at the clinic where the patient was recruited. Interviews were audio-recorded using their default smartphone software for recording and were transcribed verbatim, and field notes were documented accordingly with an audit trail. Any potential participants’ identifiers were redacted from transcripts. Each transcript was cross-checked by the research team for validation.

Analysis

Interviews were transcribed into NVivo 14 software (Lumivero, LLC, Denver, Colorado, United States) for data organisation and analysis. All transcripts were de-identified by redacting any direct identifiers to maintain confidentiality and privacy protection. 

**Figure 1:**
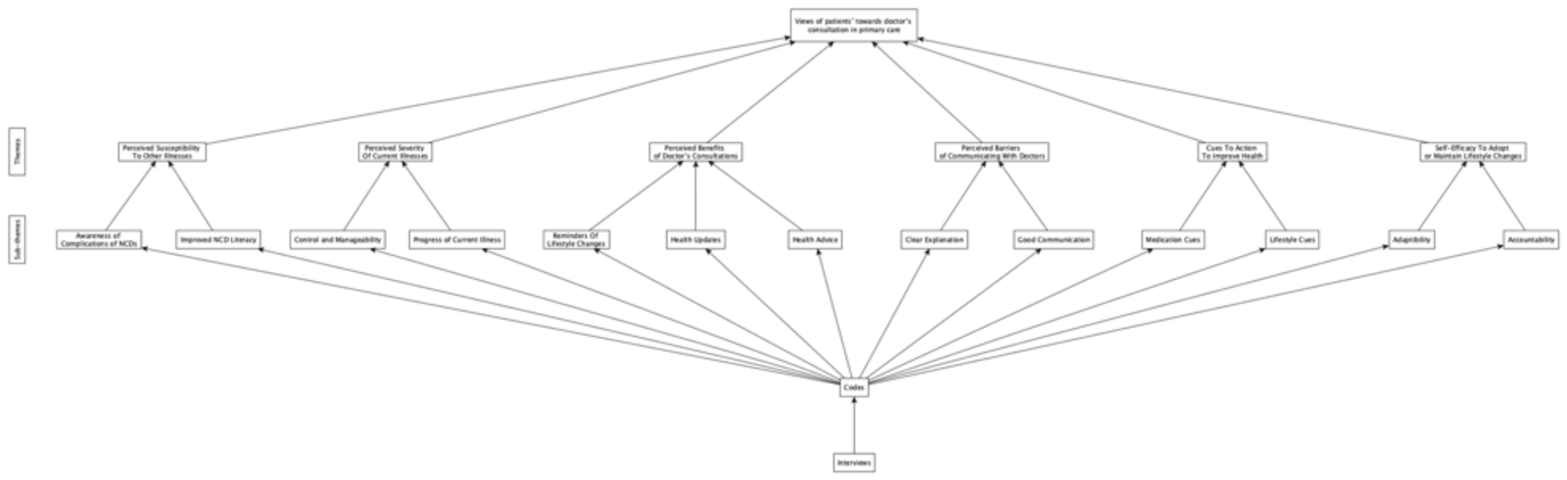
Deductive approach for views of doctors' consultations Image Credit: Authors; created with yEd software (yWorks GmbH, Tübingen, Germany)

Deductive analysis techniques were used to examine patients' views of doctors' consultations (Figure 1). The transcripts were systematically reviewed, and relevant segments of text were highlighted and assigned corresponding codes based on concepts relevant to the HBM. Codes were reviewed and grouped into sub-themes based on similarities and relationships. Sub-themes were then aggregated into broader, predetermined main themes that aligned with the HBM framework.

**Figure 2:**
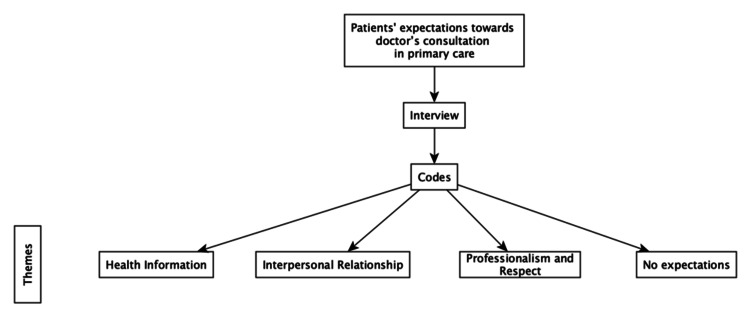
Inductive approach for ptients' expectations towards doctor's consultations Image Credit: Authors; created with yEd software (yWorks GmbH, Tübingen, Germany)

The expectations of doctors' consultations component of the interviews were analysed separately using inductive techniques (Figure 2). Codes related to expectations were identified, and new themes were developed based on these responses. This allowed for a comprehensive understanding of patient expectations distinct from other aspects of the consultation experience.

Discrepancies in coding were discussed and resolved through consensus. All classifications were decided through a consensus by the research team. Quotes that best represented the themes were chosen and tabulated to support the findings.

Language and translation

The interviews for this study were conducted in the Malay language, which was the native language of the participants. It was then analysed and coded in Malay. To ensure that the findings could be effectively communicated in the publication, the quotes used in the findings were translated into English by the bilingual authors, whose mother tongues were Malay while having a strong command of English. As Malaysians, their deep understanding of both languages and cultural contexts allowed them to accurately and faithfully convey the participants' sentiments and perspectives, while taking care to preserve the original meaning and nuances of the participants’ responses.

To further ensure the accuracy and integrity of the translations, each translation was cross-checked by the research team to minimise the potential for misinterpretation or loss of meaning. By having native speakers handle the translation, the study maintains a high level of linguistic and cultural fidelity, ensuring that the participants' voices are authentically represented in the final publication.

## Results

Participants

A total of 15 patients were recruited and participated in this study, where five participants were recruited from each of the three clinics. The majority of the participants were in the age group of 60-69 years, were male (73.3%), public servant pensioners (33.3%), and had completed secondary high school education (46.6%). The age of participants ranged from 40-74 years old, with a mean of 58.7 years. The majority of the participants were of the Malay race (73.3%). Co-morbidities were mixed; however, most of them had type II diabetes. Duration of follow-up under the NCD clinic was 1-15 years, with a mean of 5.2 years. The analysed data were divided into two parts based on the research question. A summary of participants' demographics is presented in Table 1, and detailed characteristics of each participant are given in Table 2.

**Table 1:** Demographic characteristics of the participants (N=15)

Characteristics		Values
Age (years)	Mean (range)	58.7 (40–74)
Sex, n (%)	Male	11 (73.3%)
	Female	4 (26.7%)
Race, n (%)	Malay	11 (73.3%)
	Chinese	2 (13.3%)
	Indian	1 (6.6%)
	Bumiputera	1 (6.6%)
Education, n (%)	High school	7 (46.6%)
	Certificate	1 (6.6%)
	Diploma	6 (40.0%)
	Degree	1 (6.6%)
Occupation, n (%)	Housewife	2 (13.3%)
	Pensioner	5 (33.3%)
	Businessman	2 (13.3%)
	Lorry driver	1 (6.6%)
	Factory worker	1 (6.6%)
	Car salesman	1 (6.6%)
	Driving instructor	1 (6.6%)
	Self-employed	1 (6.6%)
Duration of Follow-Up (years)	Mean (range)	5.2 (1–15)
Reason for Follow-Up, n (%)	Type II diabetes mellitus	11 (73.3%)
	Dyslipidaemia	8 (53.3%)
	Hypertension	8 (53.3%)
	Cerebrovascular accident	1 (6.6%)
	Bronchial Asthma	1 (6.6%)

**Table 2:** Detailed demographic characteristics of individual participants DM: diabetes mellitus

ID	Age	Sex	Ethnicity	Education	Occupation	Clinic	Duration of Follow-up (years)	Reason For Follow-up
P1	58	Female	Malay	High school	Housewife	Bandar Tun Hussein Onn	2	Type 2 DM, Hypertension, Dyslipidaemia
P2	74	Male	Malay	Degree	Pensioner	Kajang	15	Type 2 DM, Dyslipidaemia
P3	35	Male	Bumiputera Sabah	High school	Lorry Driver	Bandar Tun Hussein Onn	1	Hypertension
P4	61	Male	Malay	Diploma	Factory worker	Kajang	11	Type 2 DM, Hypertension, Dyslipidaemia
P5	72	Male	Chinese	High school	Car salesman	Bandar Tun Hussein Onn	1	Hypertension, Dyslipidaemia
P6	48	Male	Malay	Diploma	Self-employed	Bandar Tun Hussein Onn	5	Type 2 DM, Hypertension, Dyslipidaemia
P7	58	Male	Malay	High school	Driving instructor	Kajang	6	Type 2 DM, Dyslipidaemia
P8	68	Female	Malay	High school	Pensioner	Kajang	10	Type 2 DM, Hypertension
P9	64	Female	Indian	Diploma	Pensioner	Kajang	2	Type 2 DM
P10	71	Male	Malay	Diploma	Pensioner	Bangi	1	Type 2 DM
P11	48	Male	Malay	Diploma	Businessman	Bangi	8	Bronchial Asthma
P12	54	Male	Malay	Diploma	Businessman	Bangi	4	Type 2 DM, Hypertension
P13	66	Female	Chinese	High school	Housewife	Bandar Tun Hussein Onn	4	Dyslipidaemia, Cerebrovascular accident
P14	63	Male	Malay	Certificate	Pensioner	Bangi	5	Type 2 DM, Hypertension
P15	40	Male	Malay	High school	Security Guard	Bangi	3	Type 2 DM, Hypertension, Dyslipidaemia

Views of patients towards doctors' consultation in Malaysia's primary care

For views of patients towards doctors' consultation in primary care, transcribed interviews were sorted into codes and analysed. They were then sorted into the predetermined themes based on the HBM: Perceived susceptibility to illness, Perceived severity of the illness, perceived benefits of taking preventive measures, Perceived barriers to taking action, Self-efficacy in executing the recommended health behaviour, and Cues to action of lifestyle changes. Selected quotes were chosen based on the best representation of the themes as interpreted by the authors. As stated earlier, quotes were translated from participants’ original interview language (Malay) into English for the benefit of the global academic community and publication purposes. 

Perceived Susceptibility to Other Illnesses

Participants expressed improved views of their susceptibility to other conditions and complications after their doctor’s consultations. The sub-themes that emerged were: (i) Awareness of complications of NCDs and (ii) Improved health literacy.

Awareness of complications of NCDs: Some participants showed good understanding of the risk of complications if their current conditions remained uncontrolled, as they could specify the potential complications.

“*Usually, they will inform me of my current condition, if my diabetes is uncontrolled, my sugar is unchecked, it will affect my nerves, my eyes - and other organs. Uh, and the long-term effect of this will be dangerous. The doctor explained all of that*.”

~ P12, 54-year-old Malay male businessman with type II diabetes mellitus and hypertension.

Improved NCD literacy: Participants conveyed improved understanding of their current condition after their doctor’s consultation. Their understanding was generally superficial, but they were able to remark on how to avoid their NCD complications.

“*...It’s good. Because, the doctor will elaborate if I ask even just a little bit. If I…say it like, how should I say it, if I don’t monitor the disease, don’t control the food…control, you know control. The effect, they said, can cause me to get other…other diseases.”*

~ P8, 68-year-old Malay female pensioner with type II diabetes and hypertension. 

Perceived Severity of Current Illnesses

Only one participant described heightened perceptions of the severity of the health condition following their consultation. Others could only generalise their condition as uncontrolled or controlled. The sub-themes found were: (i) Control and manageability, and (ii) Progress of current illness.

Control and manageability: Participants were able to gauge the control of their NCD at the surface based on their doctor’s feedback and explanation.

“...Okay, usually the doctor will give reasoning, okay? For example, your sugar is high, you need to be careful or else your kidneys will be ruined, you can get other problems…so you need to control. So, I think…I am satisfied with their advice.”

~ P4, 61-year-old Malay male factory worker with type II diabetes, dyslipidaemia and hypertension.

Progress of current illness: Participants gained an understanding of why their illnesses were severe after talking to their doctor.

"...I came, and they asked how I am, what do I need today. They explained my cholesterol was high. Because of that, my blood pressure was increased a little bit.”

~ P13, 66-year-old Chinese female housewife with dyslipidemia and cerebrovascular accident.

Perceived Benefits of Doctor’s Consultations

Participants identified various benefits of their doctor's consultation. Doctor’s consultations were a medium for participants to ask questions regarding their health, as well as get updates on their health status. It also served as a prompt for them to continue their lifestyle changes. Identified sub-themes were: (i) Reminders of lifestyle changes, (ii) Health updates, and (3) Health advice.

Reminders of lifestyle changes: Most participants highlighted getting useful lifestyle advice, like food intake, exercise, and getting medications from doctors that could help manage their NCDs.

 *“...Whatever that is necessary. Reminder about food intake. Mostly about food intake. Sometimes - other things that usually the doctor talks about are about a healthy lifestyle*.”

~ P2, 74-year-old Malay male pensioner with type 2 diabetes mellitus and dyslipidaemia.

Health updates: Participants also mentioned that the consultation keeps them updated on their health condition and serves as a reminder to maintain or revert to a healthier lifestyle.

“*...Yes, because they can make us aware if our disease is controlled or uncontrolled, and sometimes the authors feel okay, but in reality, the authors are not, so when the authors know where our categories are, easy for the doctors to make decisions regarding dosage, and which part of my lifestyle that could be improved.” *

~ P11, 48-year-old Malay male businessman with bronchial asthma.

Health advice: Participants shared how doctors’ consultations provided them with practical advice to improve their NCDs.

*“...They advised me to watch my health. Eat my meals at regular intervals…and smoking - they asked me to smoke less. Because I…am a very heavy smoker.”* - P7, 58-year-old Malay male driving instructor with type 2 diabetes mellitus and dyslipidaemia.

Perceived Barriers to Communicating with Doctors

All participants reported no barriers in communicating with doctors during consultation. Participants highlighted that doctors were friendly and communicated very well during consultation, and they were given clear explanations on their queries. Sub-themes discovered were: (i) Clear explanation and (ii) Good communication.

Clear explanation: Participants mentioned that they were given detailed explanations from their doctors during consultations.

“…So far, I found the doctors are friendly. From A to Z, what should be done, what shouldn’t be done. But it’s myself that didn’t follow the doctor's advice, so I face the consequences. Doctor has already given their advice, I don’t follow, I will get the repercussions. Doctor has already given the medicine, follow their steps, but I don’t follow - no doctors can help that.”

~ P15, 40-year-old Malay male security guard with type 2 diabetes mellitus, hypertension, and dyslipidaemia.

Good communication: Participants had no trouble understanding the explanation by their doctors during consultation.

“…All the while I’ve met with the doctor, I have had no problems. I could…Communicate with them, and I understand what they are trying to explain to me.”

~ P14, 63-year-old Malay male pensioner with type 2 diabetes mellitus, hypertension, and dyslipidaemia.

Cues to Action to Improve Health 

Participants discussed various cues provided by the doctor during consultation that prompted them to initiate health behaviour change following the consultation. These cues mainly came from advice from the doctor, which came to be very effective in controlling their NCDs. Sub-themes found were: (i) Medication cues and (ii) Lifestyle cues.

Medication cues: Participants received practical advice regarding medication usage that helped them improve their NCD control.

“...The good thing is, any advice like - okay, half an hour before I do sports, take one puff. These kinds of things help me to go on with doing sports.”

~ P11, 48-year-old Malay male businessman with bronchial asthma. 

Lifestyle cues: Participants received various cues for lifestyle adjustments and improvements that could help with their NCDs.

“...Don’t adjust medications on your own. The most important thing is, people my age must do a lot of exercise. Control my food…I needed to realise that more.”

~ P6, 48-year-old self-employed Malay male patient with type 2 diabetes mellitus, hypertension, and dyslipidaemia.

Self-Efficacy to Adopt or Maintain Lifestyle Changes

Participants varied in their confidence in their abilities to successfully adopt and maintain health behaviour changes suggested during their doctor’s consultation. Most acknowledged the importance of the suggestions; however, many had factors influencing their self-efficacy, like self-motivation, self-control, and work. Sub-themes found were: (i) Adaptability and (ii) Accountability

Adaptability: Despite understanding the benefits of lifestyle changes based on their doctor’s advice, some participants found it hard to adapt and maintain the suggestions due to life commitments.

*“...I do realise what the doctor said, but sometimes I do skip my medications. I really do skip my medications sometimes. If there’s no food, or I am working, I forget to take the medications.*”

~ P5, 72-year-old Chinese male car salesman with hypertension and dyslipidaemia.

Accountability: Participants also mentioned that doctors’ consultations helped them to become more accountable for their own health, helping them make better choices.

“...The difficult part is the sweet drinks. Ha, sweet drinks. But sometimes I do control. Whenever I eat, I will remind myself - like those sweet kueh when I go to the stalls, all sugar. Apam is sweet, kaswi is sweet, so I will move towards non-sweet kueh. Like curry puffs. I seldom take the ones made of bananas now too…”

~ P10, 72-year-old Malay male pensioner with type II diabetes.

Patients' expectations towards doctors’ consultation in Malaysia's primary care

For part 2, transcribed interviews were analysed into codes. Four major themes emerged regarding participants’ expectations toward doctors’ consultation: (i) Health information, (2) Interpersonal relationship, (3) Professionalism, and (4) No expectations. Selected quotes are chosen based on the best representation of the themes as interpreted by the authors. 

Health Information

Many participants highlighted the importance of getting updates regarding their health status. They were very particular about the results of their blood investigations, as their consultation revealed their progress based on the numbers. Additionally, the participants expected lifestyle advice from the doctors on how to improve their current conditions. 

“...But I think, I hope that my…when the doctor explains - this and this is how much, what is the target, so Alhamdulillah, what they said - there is progress…”

~ P1, 58-year-old Malay housewife with type 2 diabetes mellitus, hypertension, and dyslipidaemia

*“...For example, my blood pressure was high. One hundred and eighty. Doctor, what is your advice? I want your views. How to reduce my blood pressure. What…should I eat. So…the doctor will give their advice. Okay, you shouldn’t do this and this…”* ~ P4, 64-year-old Malay male factory worker with type 2 diabetes mellitus, hypertension, and dyslipidaemia.

*“...In my heart, I was waiting for him to ask about that, because I too want to ask, if there is a problem, of course I want to know, where the problem is from Just like that…”* ~ P3, 35-year-old male Bumiputera lorry driver with hypertension.

Interpersonal Relationship

Participants expressed a preference for friendlier consultations that were clear and not stressful. They expected to leave the clinic feeling joyful, and there was a notable value placed on being treated with the warmth and care akin to that of a family member.

“...That’s why the doctor must play their role. They must want the patient, whenever they come out of the clinic - happy. That’s for me. But the one I saw, alhamdulillah, made me very happy. It felt fun!”

~ P1, 58-year-old Malay housewife with type 2 diabetes mellitus, hypertension, and dyslipidaemia.

“...It’s fun like that. From start to finish. It felt fun. When I say something, or want to ask anything, I have no fear. I understand. Because…the one doctor I saw, sometimes they tell me that their mother had the same condition like mine. So, they treat me like their mother…”

~ P8, 62-year-old Malay female pensioner with type 2 diabetes mellitus and hypertension.

“...When I talk to the doctor, I feel relieved if the doctor is friendly. When they ask, they speak softly, same when they give their advice. They ask me to monitor at home, I feel like family. I really like it; it makes me feel calm and relaxed. Makes me feel like a part of my family.”

*~* P9, 64-year-old Indian female pensioner with type 2 diabetes mellitus.

Within this theme, the authors also found that the majority of participants did not mind seeing different doctors during their appointments. Out of 15 participants, only two expressed a desire for consistent consultations with the same doctor.

“...I don’t really care; any doctor is fine. Because, to me, the doctors here are very friendly, I can communicate with them all. I’m used to it. I could communicate with them fine. I don’t care which doctors - Indians, Malay, Chinese, I don’t mind…”

~ P4 - 61-year-old Malay male factory worker with type II diabetes, dyslipidaemia and hypertension.

“...If I get to see the same doctor, it would make it easier. Meaning that I know that the advice is from one single doctor. So, it’s not convoluted. It’s not messy when…I get the advice. Now, I get different advice from different doctors…”

*~* P7, 58-year-old Malay male driving instructor with type 2 diabetes mellitus and dyslipidaemia.

*Professionalism and Respect* 

One of the core expectations discovered was respectful treatment and professionalism. Participants expected the doctor’s consultation to be professional, while respecting their concerns and secrecy.

“...For me, I hope doctors watch their professionalism. They have their ethics, doctor’s ethics, right? So don’t deviate from their ethics. That’s what it’s supposed to be. I don’t have to explain it but, there are things they should really maintain, like secrets of the patients…”

~ P12, 54-year-old Malay male businessman with type 2 diabetes mellitus and hypertension.

“...Yes, they respect me, I will respect them. Mutual respect, between two people. That’s it.” 

~ P4, 61-year-old Malay male factory worker with type 2 diabetes mellitus, hypertension, and dyslipidaemia.

No Expectations

Some patients reported having no specific expectations for their consultations. They believed the doctors they saw have served them well throughout the years of follow-ups.

“...Ah, I don’t think there are changes necessary. For me, until this second, everything is okay, good.”

~ P2, 74-year-old Malay male pensioner with type 2 diabetes mellitus and dyslipidaemia.

“No changes, everything is good. The doctors are good. The services are okay.”

~ P13, 66-year-old Chinese housewife with dyslipidaemia and cerebrovascular accident.

## Discussion

This qualitative study aimed to explore and understand two main aspects of patients' experiences with doctor consultations in primary care: their perceptions of these consultations and their expectations from them, using the HBM as a theoretical framework. The findings offer a comprehensive understanding of how patients perceive various elements of their doctor’s consultation experiences and how they have affected their lifestyle, as well as their expectations of future consultation visits.

Patients' perception of doctors’ consultation in primary care

Analysis revealed that participants had an overwhelmingly positive perspective of doctors’ consultation in primary care. Participants viewed doctors’ consultation as modifying their perceived susceptibility, perceived severity, and perceived benefits of their NCDs, especially in the domain of lifestyle changes. These consultations were found to serve as crucial reminders for patients to maintain or revert to healthier lifestyles. This provides a generally optimistic view on how consultations were conducted at the primary care setting in Malaysia. Similarly, most patients in the United Kingdom view their general practitioners (GPs) as a credible source of health information, and are well placed to offer behaviour change interventions [13]. This also echoes a study done in Malaysia that highlighted patients’ preference for getting advice from doctors rather than nurses, as they perceived doctors to be more knowledgeable [14].

In relation to that, health literacy has long been researched as a positive predictor of improving overall health. WHO has developed a guideline for health literacy development [15], highlighting its importance in controlling and preventing NCDs. This study demonstrated that patients had an understanding of their diseases as they were able to identify the severity of their NCDs and articulate the potential risks and complications associated with conditions such as diabetes and hypertension. Despite that, while some could specify the complications, most participants were only able to provide generalised explanations of the risks. This could reflect the surface depth of their insight, which is affected by various determinants like geographic areas and education level, as shown by previous studies [16]. Nonetheless, an objective measurement of participants’ health knowledge would be needed to determine the significance, as recent evidence suggests that East Asian NCD patients had limited health literacy [17].

Furthermore, the authors discovered that there were no barriers faced by the participants during their doctor consultations. This may lead to participants being more open to asking questions to their doctor. In addition to that, participants often described the doctors as friendly and effective communicators who provided clear explanations. The authors found this surprising, as this finding went against international studies that highlighted some common barriers faced by patients when communicating with doctors, including time constraints, doctors’ attitude, privacy, cultural and racial differences, linguistics, and use of medical jargon [18]. A possible explanation of this finding is that the participants had been going for multiple consultations throughout the years of follow-ups, leading to adaptation to the consultation patterns.

It was also evident that doctors' advice during consultations acted as significant cues to action for the participants, prompting them to initiate and maintain health behaviour changes. They cited specific advice, such as exercise and dietary recommendations, as vital changes needed in their lives to control their conditions. This suggests the participants have been getting appropriate and acceptable suggestions from their doctors. Previous studies have shown that the role of actionable advice in motivating patients towards healthier behaviours may help patients overcome the barriers of managing their NCDs [19]. Nevertheless, it is important to further research the sustainability and effectiveness of these cues to actions.

While most participants acknowledged the importance of the doctors' advice, the efficacy of patients to act on the recommendations was found to be polarising. Factors such as self-motivation, self-control, and work commitments were highlighted to influence their adherence. For instance, some participants struggled with medication adherence and dietary control, highlighting the need for additional support to enhance and facilitate continuous behaviour changes. This supported the findings of a Malaysian study by Nor et. al., which identified self-efficacy, motivation, knowledge, habits, social support, socioeconomic, sociocultural, and time management as barriers to making lifestyle changes among type 2 diabetes mellitus patients [20].

Patients' expectations of doctors' consultation in primary care

The second part of this study explored patients' expectations of doctor consultations in primary care. This study found that the majority of participants expected their doctors to be a credible source of information about their health during follow-ups. This resonates with a study done in Spain, which highlighted that patients placed the utmost importance on getting health advice and explanation from their doctors [21]. 

Notably, participants placed high importance on receiving detailed updates about their health status, particularly regarding blood test results. They expressed a clear desire for doctors to explain these results comprehensively and offer specific lifestyle advice to manage their conditions. A study on general practices in the Netherlands explained this behaviour: patients believe biometric data are concrete proof of their health status, as well as a tool for the detection of complications that could be acquired from their NCDs [22]. This may reflect a proactive approach where patients seek to understand their health metrics and how to improve them.

A literature review by Deledda et al. summarised 27 international papers studying what patients expect from doctors’ communication and came to the conclusion that “Physicians are expected to be friendly, respectful, interested, non-judgmental and sensitive and to treat patients as a person and as a partner” [23]. It is important to note that the author accepted that the concept of patients' expectations is abstract and could differ based on the focus of inquiry. However, when asked about expectations for physicians themselves, patients highlight relationship-centred behaviours.

Likewise, this research revealed that participants expect doctors to be friendly and approachable, so that the consultations are not stressful and they can get more health-related information. They expected consultations to be friendly, clear, and not stressful, with a strong emphasis on leaving the clinic feeling happy and relieved. This puts punctuation on the importance of good communication and empathy in consultations, as a recent survey done on Americans revealed that 48% of adults reported being anxious prior to their doctor’s appointments [24].

In addition to that, participants also had strong expectations for doctors to exhibit high levels of professionalism and to treat patients with respect and dignity during consultation. While the definition of professionalism is abstract, it has been linked to increased empathy and altruism among doctors [25]. The significance of professionalism and respectful treatment identified in this study aligns with a systematic review that demonstrated patients who felt respected and valued by their doctors reported better overall experiences and outcomes [26].

Interestingly, a notable subset of patients reported having no specific expectations for their consultations. This could reflect a high level of trust in doctors in Malaysia's primary care system or participants’ more passive approach towards their health. An alternative explanation would be participants’ more passive approach regarding their health. 

A fascinating discovery of this research contradicted numerous studies that put continuity of care as a priority for patients [27]. Only two participants highlighted wanting to see the same doctor for every follow-up. The authors followed up with 13 other interviews asking this specific question; however, the rest of the participants did not find seeing the same doctor important. This may be attributed to technological advancements like electronic medical records (EMR), where a patient's complete medical history, current treatment plans, and any previous notes from other doctors are documented and accessible by any authorised healthcare provider. This may also signal that most participants prefer accessibility, as they do not need to wait for appointments to see the same doctors, especially in Malaysia’s current primary healthcare system, where patients are rarely allowed to choose their doctors.

Strengths

The qualitative approach enables an in-depth exploration of patients' views and expectations, as well as capturing the subjective aspects that may be missed in quantitative studies. The authors have taken steps to ensure rigour, trustworthiness, and credibility of this study. As outlined in the methodology, the authors have adapted the framework outlined by Lincoln and Guba, which includes four criteria: credibility, transferability, dependability, and confirmability [12]. 

The application of the HBM provides a structured and comprehensive framework that has allowed us to undergo a detailed and coherent exploration of the complex dimensions of a patient's lived experiences, while fulfilling the objectives of the research. This corroboration with an established and well-understood theory made analysis more systematic and adds to the robustness of this study.

IDI was chosen as the data gathering tool of choice, and this has helped the interviewers to build rapport and trust with the participants. This participant-centred approach translated their complex experience into detailed narratives and direct quotations, resulting in rich and vivid insights that enhanced the validity and reliability of the findings.

Limitations

The authors acknowledge the limitations of the study. The sample size (n=15) was achieved after no new themes were yielded during the final few interviews, suggesting sufficient sampling. While the authors aimed for maximum variation sampling, it is important to note that 73.3% of the respondents in the final samples were male, and the authors recognise the lack of racial diversity. This falls under selection and researcher bias, and this may skew the findings when certain demographics are underrepresented. In the authors’ defence, this demographic distribution reflects the purposive nature of the sampling strategy, where participants were selected based on their ability to contribute valuable information to the study, rather than to achieve a perfectly balanced sex or race representation. In context, male and Malay participants have been more readily available or more willing to participate in interviews. 

Moreover, the achievement of data saturation with this sample composition suggests that the key themes were sufficiently captured despite the gender and racial imbalance. While a more balanced sample might have provided additional perspectives, the data collected were sufficient to meet the research objectives. 

The age group of participants was also found to be older, as NCDs are known to affect the elderly population more. This contrasts with the more diverse socio-demographics of Malaysia, making the possibility of findings being geographically context-specific, thus generalisability cannot be assumed. An age-group-focused sampling may provide further insights. 

Participants were also recruited from the NCD clinics, where continuity of care is established as they have undergone follow-ups for at least one year. The sample, therefore, is unlikely to represent the full spectrum of patients coming to government primary clinics, as different sets of patients, for example, coming for acute illness, will be seen by doctors in the outpatient unit. However, homogeneity of the co-morbidities, age, and years of follow-up strengthens the findings. 

In addition to that, participants were also informed that they were interviewed by doctors. This could have influenced the responses as patients may hesitate to describe negative experiences to a healthcare professional directly, although they were all informed to freely express their views without prejudice. The authors postulate that different perspectives may be gained if the interviewers were not medical personnel. Nevertheless, the interview questions were constructed based on HBM as the theoretical framework reinforces the rigidity of this study. In spite of that, themes outside of the framework may materialise if participants were interviewed in another method, such as focus group interviews, where participant-to-participant interactions may encourage further experience sharing and thus provide novel insights.

Implications for practice

This research fills important gaps by providing a nuanced understanding of patient perceptions and expectations in primary care consultations. The authors believe this research has provided valuable points of view that could help healthcare workers and policymakers to strategise in enhancing patient satisfaction, engagement, and health outcomes in primary care settings. The positive perceptions identified provide a holistic view of how well the doctors have been communicating with the participants. The expectations of a doctor’s consultation provided valuable insights on how patients want to be treated. Existing literature often focuses on technical and procedural aspects of healthcare [28], and this research has investigated the critical role of the emotional and relational aspects of healthcare, which are less frequently explored in depth. 

Beyond the emerging digital trends like telemedicine [29] and artificial intelligence [30], this study provides a counterpoint to the growing body of literature on digital health, reminding healthcare workers and policymakers of the enduring importance of traditional consultation elements that go hand-in-hand with clinical competency and are not comparable with machines. 

Recommendations

Recommendations for Policymakers

Based on the findings, the authors recommend continuous education and training for healthcare providers focusing on effective communication and interpersonal skills during consultations to ensure patients’ expectations are met. This is evident by studies done internationally where training doctors in communication skills improved their patients’ satisfaction, and they perceived trained doctors to be more interested and friendly, while the effects and benefits are still seen in the long term [31]. In addition to that, the authors found that patients perceived doctors as a credible source of information regarding health. It is therefore important to push for frequent courses that update doctors regarding the latest medical knowledge so that they can impart correct and current knowledge to their patients. 

Moreover, resources should be allocated for primary care to establish a universal EMR system, to establish a continuity of care despite the high turnover of doctors. This is to align with findings that theorize patients may prefer access and continuity that could be provided by EMR, rather than seeing the same doctors.

Lastly, a continuous survey of patients’ satisfaction regarding doctor’s consultation may help alter policies as the medical field is evolving along with the patients' views and expectations. Patients' direct feedback has been shown to improve doctors’ consultation skills as well [32].

Recommendations for Future Research

A large-scale, quantitative, longitudinal study with a larger sample size and more diverse patient demographics may examine how patients' views and expectations evolve over time, particularly in response to changes in healthcare practices and the utilisation of technology. As these findings are affected by cultural, socioeconomic, and geographic factors, the authors recommend further qualitative studies at different areas in Malaysia to understand views and expectations of patients in different contexts.

## Conclusions

This study revealed that patients had a positive view of Malaysia’s primary care doctors’ consultations. These clinic visits helped in improving their perceived susceptibility, perceived severity, and perceived benefits of their NCDs. However, there is room for improvement in enhancing patients' perceptions of severity and supporting their self-efficacy. This paints an optimistic outlook of the healthcare system in general.

Key expectations that patients had for their doctors’ consultation were comprehensive health information, empathetic interpersonal interaction, and professionalism. There is a subset of patients who had no specific expectation, which may indicate complete trust in their doctors or a more passive approach towards their health. These insights could form strategies for doctors and policy-makers to enhance engagement with patients, and further empower them in managing their health conditions through improving health literacy regarding their conditions, and provide sustained support.

## Appendices

**Table 3:** Interview Guide

Introduction	Explain to the informant that you are doing a project to understand patient’s perspective on doctor’s consultation
	Explain that you will be asking their experiences during their latest consultation with a doctor. You want them to be as open and honest when answering
	Ensure confidentiality
	Remind them that the interview will be recorded, transcribed verbatim and analysed
	All information collected will be kept safe and secret.
	You will not use their name or any identifying information and everything they say will be used only for research purposes
	Remind them of their right to stop the interview whenever they want
	Explain to the informant that you are doing a project to understand patient’s perspective on doctor’s consultation
Guide and Prompts	Hi, I am Dr. X, currently working in XXX. How are you today?
	Why did you come to the clinic on XX/XX/XXXX?
	May I know what was your expectation before meeting with the doctor?
	What did you get from that consultation?
	Did the consultation met your expectation?
	What were the good points of the consultation?
	What were the bad points from the consultation?
	What do you hope for improvements in future doctor’s consultation?
	Thank you for participating. Can we contact you again if I have further questions or I need further clarification?
